# Contextual factors and implementation strategies for a multi-level community-based sodium reduction intervention in Chicago’s South Side: a qualitative study

**DOI:** 10.1186/s12966-025-01794-9

**Published:** 2025-07-06

**Authors:** Olutobi A. Sanuade, Allison J. Carroll, Ricky Watson, Jiancheng Ye, Jennie L. Hill, Jonathan Chipman, Fernando A. Wilson, Andy J. King, Abel Kho, Guilherme Del Fiol, Paris Davis, Justin D. Smith

**Affiliations:** 1 Department of Population Health Sciences, Division of Health System Innovation and Research, Spencer Fox Eccles School of Medicine at The University of Utah, Salt Lake City, UT USA; 2 Department of Psychiatry and Behavioral Sciences and Center for Dissemination and Implementation Science, Northwestern University Feinberg School of Medicine, Chicago, IL USA; 3 Center for Health Information Partnerships, Institute for Public Health and Medicine, Northwestern University Feinberg School of Medicine, Chicago, IL USA; 4 Weill Cornell Medicine, Cornell University, New York, NY USA; 5 Cancer Control and Population Sciences Program, Huntsman Cancer Institute, University of Utah, Salt Lake City, UT USA; 6 Matheson Center for Health Care Studies, University of Utah, Salt Lake City, UT USA; 7 Department of Communication, University of Utah, Salt Lake City, UT USA; 8 Department of Biomedical Informatics, University of Utah, Salt Lake City, UT USA; 9 Total Resource Community Development Organization, Chicago, IL USA

**Keywords:** Sodium reduction, Hypertension, Community-based intervention, Barriers, Facilitators, Implementation strategies

## Abstract

**Background:**

Excessive sodium intake exacerbates rates of hypertension. African American adults have higher rates of hypertension in part due to a higher-sodium diet. The multi-level Communication for Behavioral Impact for Sodium Reduction (COMBI-SR) community-based intervention effectively reduces sodium intake in international settings, but it has not yet been implemented and tested in the U.S. This study explored the contextual factors (barriers/facilitators) and implementation strategies for COMBI-SR in Chicago’s South Side neighborhood–an area with high rates of hypertension.

**Methods:**

Between May and November 2023, we conducted qualitative interviews with potential intervention recipients (*n* = 8), Research Ministry Ambassadors (*n* = 5) and healthcare professionals (*n* = 2), 1 focus group with potential intervention recipients (*n* = 9) and 3 focus groups with healthcare professionals (*n* = 10). The Consolidated Framework for Implementation Research (CFIR) 2.0 guided the development of semi-structured interview guides. Thematic analysis was performed using CFIR 2.0 constructs to identify barriers and facilitators to implementation, and the Expert Recommendations for Implementing Change (ERIC) compilation to identify implementation strategies.

**Results:**

Key barriers included a lack of awareness of sodium content in foods, socioeconomic disparities limiting access to healthy options, and cultural dietary traditions. Facilitators included strong community partnerships, engaged faith-based organizations, and openness to integrating technology, such as a mobile app, to help monitor and reduce sodium intake. Specific strategies to support sodium reduction involved simplifying public health messages, offering low sodium cooking demonstrations, promoting healthier food options through community outreach, and providing personalized education on reading nutrition labels and managing sodium intake.

**Conclusions:**

Successful implementation of COMBI-SR in Chicago’s South Side requires addressing financial, educational, and cultural barriers while leveraging trusted community structures to promote sustainable sodium reduction. These findings will guide future efforts to implement COMBI-SR in the U.S., emphasizing culturally tailored messaging and ongoing community engagement to improve cardiovascular health.

**Supplementary Information:**

The online version contains supplementary material available at 10.1186/s12966-025-01794-9.

## Background

Almost 1 in 3 American adults has hypertension [1]. About three-quarter of U.S. adults with hypertension have uncontrolled blood pressure (BP), increasing their risk for heart disease and stroke [1]. Hypertension contributes to 685,875 deaths annually in the United States (U.S.), and disproportionately affects African American (AA) adults [2]. The 30-year gap in life expectancy between Chicago’s South Side predominantly AA neighborhoods, for example, and the northern, predominately white neighborhoods is partly attributable to hypertension rates [3, 4]—78.7% in the South Side compared to 21.7% in the northern neighborhoods [5].

 Excess sodium intake is associated with high BP and increased risk of heart disease and stroke [ 6, 7 ]. The largest number of diet-related deaths globally (1.89 million deaths) is attributed to excess sodium intake [ 8 ]. The American Heart Association (AHA) recommends sodium intake of < 2300 mg/day to reduce risk for cardiovascular disease [ 9 ]. Average daily sodium intake among AA adults exceeds 4,500 mg/day, with > 80% exceeding the recommended sodium intake level [ 10 ]. Reducing sodium intake could prevent thousands of deaths annually, significantly reduce the prevalence of high BP, improve cardiovascular health, and save $18 billion/year in healthcare costs [ 11 ].

Communication for Behavioral Impact for Sodium Reduction (COMBI-SR) is a multi-level evidence-based intervention (EBI) designed to empower people to improve their diets and increase demand for lower-sodium food products [12]. COMBI-SR includes both individual- and community-level approaches. At the individual level, tools like the SaltSwitch smartphone application (app) help users change their behaviors and decrease personal sodium intake [13]. At the community level, mass media communication campaigns, community outreach, and partnerships with local healthcare and government organizations drive shifts in culture surrounding sodium knowledge and intake. Previous trials showed that COMBI-SR effectively reduced sodium consumption, lowered BP, and improved knowledge and behaviors regarding sodium consumption [12, 14, 15]. These studies took place in Vietnam and Australia; thus, the intervention and implementation need adaptation to local contexts to be effective and sustainable in the U.S.

Understanding contextual determinants (i.e., barriers and facilitators) and implementation strategies is crucial for the successful adaptation, adoption and sustainability of EBIs like COMBI-SR [16, 17]. Exploring barriers and facilitators allows researchers to develop targeted strategies to overcome challenges and leverage strengths within the community. Moreover, using a framework-guided approach not only provides structure for inquiry and analysis, but is also more likely to result in generalizable knowledge for others. Determinants frameworks, such as the Consolidated Framework for Implementation Research (CFIR 2.0) [18], are used to understand and analyze barriers and facilitators to implementing and sustaining an EBI. The Expert Recommendations for Implementing Change (ERIC) study produced a comprehensive compilation of strategies to facilitate identification of actionable strategies that enhance adoption and sustainability of EBIs [19]. This approach supports the development of adaptable and implementable EBIs for diverse communities, while also improving their effectiveness and scalability.

This study explores the contextual factors and strategies for implementing the COMBI-SR program in Chicago’s South Side neighborhood. The findings will guide the development of context-specific adaptations of COMBI-SR for this community, ensuring the intervention is effective, adopted, and sustained.

## Methods

### Study design and settings

A qualitative study was conducted to understand barriers and facilitators to reducing sodium intake (i.e., salt use during cooking, salt use at the table, selection of low salt processed foods) and implementation strategies for addressing these determinants in Chicago’s South Side neighborhood. The South Side has a predominantly AA population, which experiences significant health disparities, including the highest rates of obesity, diabetes, hypertension, heart disease, and stroke in the city [20]. Within this community, faith-based organizations (FBOs) serve as influential and trusted institutions for health-related education, research, and advocacy [21, 22]. This study was reported according to the Consolidated Criteria for Reporting Qualitative Research (COREQ) (Supplemental Material 1). This study was approved by Northwestern University’s Institutional Review Board (STU00212622).

### Study participants

Participants aged 18 years and above with diverse perspectives on context and strategies for sodium reduction were purposively sampled from Chicago’s South Side community. Recruitment was conducted in partnership with Pastors for Patient-Centered Outcomes Research (Pastors4PCOR), a Patient-Centered Outcomes Research Institute (PCORI)-funded community health outreach initiative that engages a diverse range of faith communities in Chicago’s South Side and south suburbs to foster partnerships between community members and researchers to address health disparities. We also worked with AllianceChicago, a health center-controlled and practice-based research network of over 50 organizations committed to advancing health equity and fostering innovative collaboration. Participants received incentives for their time: $150 giftcards for potential intervention recipients and research ministry ambassadors, $250 giftcards for healthcare professionals.

#### Potential intervention recipients

We recruited adult community members living with hypertension and/or caring for someone with hypertension from FBOs on the South Side of Chicago.

#### Research ministry ambassadors (RMAs)

RMAs are leaders in Chicago’s South Side faith-based communities who underwent research and community engagement training with Pastors4PCOR. They facilitate engagement across diverse faith communities and offer researcher workshops on strategies and methods for engaging both academic and medical research communities.

#### Healthcare professionals

We recruited healthcare professionals (physicians, nurse practitioners, community healthcare workers, pharmacists, clinic administrators, and physician assistants/primary care providers) from AllianceChicago Federally Qualified Health Centers (FQHCs) clinics located in Chicago’s South Side.

### Interviews and focus groups

Data were collected though semi-structured interviews and focus groups between May and November 2023 by three authors with qualitative research experience (OAS, JY, RW). Interview and focus group participants were unique individuals, ensuring a broad range of perspectives and experiences were captured. Fifteen semi-structured interviews (approximately 30 min each) were conducted with potential intervention recipients (*n* = 8), RMAs (*n* = 5) and healthcare professionals (*n* = 2). Four focus groups (approximately 90 min each) were conducted, including 1 group of potential intervention recipients (*n* = 9) and 3 groups of healthcare professionals (*n* = 10). All eligible participants agreed to participate.

Semi-structured interview guides were developed using CFIR 2.0 [18], and modified based on discussions with Pastors4PCOR leadership. To facilitate discussion, participants were shown visual and educational messaging on sodium intake from Centers for Disease Control and Prevention and the World Health Organization and a demonstration of the SaltSwitch app. The focus areas for interview guides by participant group are outlined in Table 1. The interview guides are provided in Supplemental Material 1. All interviews and focus groups were conducted in English and recorded via the Zoom videoconferencing platform.

**Table 1:** Focus areas for interview guides by participant group

Participant Group	Focus Areas
Potential Intervention Recipients	• General attitudes towards sodium consumption. • Awareness of daily sodium intake recommendations.
Research Ministry Ambassadors	• General attitudes towards sodium consumption. • Awareness of daily sodium intake recommendations. • Effectiveness of public health messaging on sodium. • Familiarity with and use of technology platforms for nutrition information. • Health impacts of excessive sodium consumption. • Willingness to promote salt reduction. • Past advocacy efforts • Potential champions for COMBI-SR • Community resources needed for successful implementation
Healthcare Professionals	• Barriers to and facilitators of sodium reduction. • Strategies for reducing sodium intake. • Role of healthcare professionals in addressing excess sodium consumption in their patient population

### Data analysis

All interviews and focus groups were transcribed verbatim, redacted for confidentiality, and uploaded to Dedoose v9.0.17 software for coding and analyses [23]. Thematic analysis was conducted following Braun and Clarke’s [24] six-phase approach. First, we engaged in data familiarization by reading through the transcripts and documenting initial impressions. Second, initial coding was performed by two authors (OAS and AJC), who collaboratively developed the codebook by first double-coding 3 transcripts (1 RMA interview, 1 FBO focus group, 1 healthcare professional focus group) to agree on key themes and optimize inter-coder reliability. Remaining transcripts were divided between the two coders for independent coding and were cross-checked and discussed. Codes were collated into potential themes and iteratively reviewed and refined to ensure they meaningfully captured patterned responses across the data. Themes were subsequently defined and named to reflect their central organizing concepts.

Although our coding was primarily inductive, we used a deductive approach to map developed themes onto CFIR 2.0 [18] to identify barriers and facilitators to COMBI-SR implementation and onto ERIC [19] to identify relevant implementation strategies. Notably, only healthcare professionals were specifically queried about strategies and coded per ERIC. In discussions with Pastors4PCOR, their preference was for potential intervention recipient and RMA interviews/focus groups to focus on contextual and cultural factors related to sodium consumption. However, many consider the Process domain of CFIR to represent implementation strategies [19] and several Process constructs were coded in the data across groups. Global themes were generated to reflect cross-cutting insights across stakeholder groups. Finally, we developed thematic networks and summary tables with illustrative quotes and a code-application matrix to show how codes aligned with CFIR 2.0 framework and ERIC compilation. Participant characteristics and survey responses (healthcare professionals only) were summarized as frequency and percentage.

### Reflexivity statement

 Our research team included both female (*n*  = 3) and male (*n*  = 9) researchers, most of whom held PhDs and had qualitative research experience. We recruited participants through existing community and clinical partnerships. A member of the community (RW) was trained to conduct interviews/focus groups with potential intervention recipients and RMAs, while interviews/focus groups with healthcare professionals were led by researchers (OAS, JY). Data analysis was led by researchers and themes and interpretations were confirmed with community partners. We were mindful of ethical implications and potential biases throughout the study. To compensate participants for their time and contributions, we provided gift cards. However, we acknowledge potential concerns, such as the risk of coercion and the disparity in amounts given to intervention recipients and RMAs ($150) versus healthcare professionals ($250), which reflected differences in time commitments and roles and was consistent with our approach in prior studies. To mitigate potential biases, we engaged in regular team meetings and discussions with our partners to reflect on the impact of the research process.

## Results

### Participant characteristics

 Table  2 provides the characteristics of the study participants. Of the 34 participants interviewed, 70.6% were female, and 70.6% identified as Black/African American. All potential intervention recipients and RMAs were Black/African American while Healthcare professionals were 66.7% White. The mean age of healthcare professionals was 45.3 years (SD = 7.9), 91.7% held a college or postgraduate degree, and 72.7% reported annual household income of $100,000—$199,999.

**Table 2:** Participant characteristics

	Total (*N* = 34)	Potential Intervention Recipients (*n* = 17)	Research Ministry Ambassadors (*n* = 5)	Healthcare Professionals (*n* = 12)
	*n* (%)	*n* (%)	*n* (%)	*n* (%) or M (SD)
**Sex**				
Male	10 (29.4)	8 (47.1)	1 (20.0)	1 (91.7)
Female	24 (70.6)	9 (52.9)	4 (80.0)	11 (8.3)
**Race**				
Asian	1 (2.9)	0 (0.0)	0 (0.0)	1 (8.3)
Black/African American	24 (70.6)	17 (100.0)	5 (100.0)	2 (16.7)
White	8 (23.6)	0 (0.0)	0 (0.0)	8 (66.7)
Other	1 (2.9)	0 (0.0)	0 (0.0)	1 (8.3)
Age, *years*	-	-	-	45.3 (7.9)
**Education**				
< College	-			1 (8.3)
College/postgrad	-			11 (91.7)
**Annual household income**				
<$100,000	-	-	-	2 (18.2)
$100,000-$199,999	-	-	-	8 (72.7)
$200,000+	-	-	-	1 (9.1)

Potential Intervention Recipients and Research Ministry Ambassadors were not asked to report age, education and annual household income

### Overall context of sodium consumption

All participants viewed excess salt or sodium as harmful, associating it with negative health conditions such as high BP, headaches, and body swelling. Many admitted to being unfamiliar with sodium consumption guidelines and did not know the recommended daily intake. Some RMAs and potential intervention recipients were unaware that sodium is a nutrient, and many associated it exclusively with processed foods, junk food, and fried items. However, respondents who self-identified as fitness enthusiasts saw sodium as essential for energy and workout performance and a few potential intervention recipients noted that certain types of salt could cure headaches. Many participants saw sodium as a flavor enhancer, making it a staple in many households for seasoning and taste.

Cultural and socio-economic factors further complicate the context of sodium consumption on Chicago’s South Side. For instance, faith-based communities often view salt symbolically, associating it with preservation and spiritual responsibility, and also as a form of punishment. Moreover, socio-economic disparities limit access to healthier, low-sodium food options, forcing many community residents to rely on cheaper, processed foods that are high in sodium. The convenience and affordability of fast foods also contribute to high sodium consumption, as busy lifestyles and limited resources make it difficult to prioritize healthier eating habits.

### Determinants of implementing COMBI-SR

The following results are presented per the five CFIR 2.0 domains: Innovation, Outer setting, Inner setting, Individuals, and Implementation Process. A summary of the constructs, corresponding themes and quotes are presented in Table 3. Details of the codes applied are presented in Supplemental Material 2.

**Table 3:** Summary of barriers and facilitators, per the consolidated framework for implementation research (CFIR) 2.0, to implementing the COMBI-SR program in chicago’s South side community

CFIR 2.0 Domain and Construct	Summary of themes	Illustrative quotes
Innovation		
Innovation evidence-base	Further research is desired to better understand the biological effects of and individual differences in salt consumption.	• As it relates to biology as it relates to food, we still need a hundred more, a hundred to 400 more years to really speak accurately about like hat salt is really doing in someone’s body, instead of just going with the hypothesis like, Oh, this person eats too much fried chicken with salt, and that’s the reason why they developed this (PIR 4, IDI) • And like me as somebody who’s passionate about fitness like have a pretty intense like workout regimen. So generally I have to consume more salts, cause that’s like what’s recommended because you’re sweating so much…So I was wondering [how that number was reached] like what factors were taken into account about like lifestyle, or is that on average I was just more curious about where the number came from. (PIR, FG)
Innovation Relative Advantage	The SaltSwitch app has the potential to be better than other available innovations, including other health-related apps.	• How is this gonna help me?… Something a lot of people say, is, “is this gonna help me lose weight,” like, you know, “How is it gonna help me if I use this app?”…It gives me a better alternative of what product I should buy instead of that one, you know. So with the having less salt intake, is it going to refer me to something that would taste good? (RMA 5, IDI) • A friend of ours started a Facebook group that we are part of called, “It’s a wrap.” And it’s like it’s just overall wellness. And it’s like motivation…Also, just trying to hang with my young adult kids, you know, they always doing something and so I get out there with them. Try to walk, walk with them, you know. (RMA 1, IDI)
Innovation Complexity	It is important to simplify the messages of COMBI-SR and the accessibility and presentation of the SaltSwitch app.	• The way nutritional information is written is confusing. You might think the listed amount is for the whole item, but it’s often for a single portion. A box might have 7 or 8 portions, so you have to multiply to figure out the total salt content (PIR 5, IDI) • Make [the app] easily accessible for the, you know, because the people can’t - like they don’t fool…They’ll be like, Okay, I just can’t do it. It’s gotta be something they can access easily. And it be just plain, simple language. (RMA3)
Innovation Design	The COMBI-SR messages and SaltSwitch app are well designed and packaged, and should be continuously improved.	• I really love the screen you showed when you could scan something on your phone. It shows all the content of the sodium. And this, that and the other. So, yeah, that’s huge. (RMA 1, IDI) • In the moment it might be good…So you’re in the store. These are like the better products here. It would need to be developed more like telling you where to go; like what’s the next best thing. But for like a startup, I think it’s a good for the benefits later. (PIR 4, IDI)
Innovation Cost	People will use the SaltSwitch app if affordable/free.	• It’s something that I feel like I will use. But honestly, I feel like there’s already apps on the market like that. I’m just too cheap to purchase them. So if it were free and you know readily available, I would be more apt to use it.(PIR 6, IDI) • I think people of all demographics like the word free. So if there isn’t a cap where you can provide services for people to get them to get them going not to be a crutch, though - cause we don’t wanna baby nobody for the rest of the walk in life. (RMA 1, IDI)
Outer Setting		
Local attitudes	Salt symbolizes enhancement, in that it enhances gatherings and the cultural significance of food practices that transcend generations.	• Salt, to me, symbolizes coming together and family. When it’s sprinkled on food, it enhances the taste and brings comfort, often drawing more people to gather around during events, gatherings, or meals. Biblically, I see it as a metaphorical meeting place, signifying a place of connection and community. (RMA 1) • I believe we season our food, and I think over time, generational, it became just a habit, not even tasting the food to see if it tastes good, we automatically always add, and I think that over time we’re not as healthy…All that sodium over, the years passed down from your grandmother and uncle, and your and our ancestors. (RMA 1)
Local Conditions	The neighborhoods have food deserts and there are multiple challenges to residents accessing healthier, low-sodium foods.	• There is no food places, not even McDonalds. So this isn’t just a food desert for anyone who don’t have a transportation. It’s almost impossible to buy food from outside unless they cook or food of their own, you know. (HP, FG 2) • I think one of the issues with foodbank is they can provide shelf stable products, right? But a lot of that is not necessarily healthy. Like I think they do the best they can…it’s whatever people donate, right? (HP, FG 2) • I feel like even my toothpaste has salt in it. It makes me wonder what’s really going on. Are we being set up for challenges in our communities and lives? It feels like we might have been sold a lot. It’s like there’s something deeper at play here, because we’re told we should only have one teaspoon of salt… who even realizes this? (PIR, FG) • And then you also have factors of like, food deserts and like safety and transportation. So like your fresh food is far, you have to get it from A to B, not only is that fresh food more expensive, it’s more expensive to go get it, and then you might not feel so safe doing that. (HP, FG1) • And I will add to like healthy, especially in the food area, like healthier foods generally are more expensive. I grew up in a low-income community. So we had a very health-conscious family, so to his point, like the healthier, like options with like organic food and all that weren’t in the neighborhood I grew up…So we had to do our best with, like the all these Jewel Oscos, you know, and just kind of pick and choose what we could find from there. (PIR, FG1)
Partnerships & Connections	A variety of existing support systems and services exist to reach a broad swath of the population, into which promotion and distribution of healthier food options could be integrated.	• It could be something that we, well, I don’t know we could provide to shelters and just kind of sneak it in and encourage them to try it. Most of the time they’re going to reach for, they don’t want to think about those things…if you just provide it and just kind of suggest, hey, try this, you know, um, try this instead. Maybe that would work. I don’t know. (HP, FG 2). • The school system would be the quickest way. Because children are impressionable, like you know, they kind of soak up anything that they learn, and if it was such a mandate or a standard in the school system. I think that that will kinda trickle over into the home and then the child is able to kind of influence the parent. (PIR 6, IDI) • The South Side has the highest net density, like more churches than any other community in the US per population. So going into the churches which are trusted spaces to work with pastors, to work with church kitchens, thinking about where are the places where people come together to cook together…The main distributors of food knowledge would be where communities come together to eat and figuring out where those spaces are. (HP, FG 1).
Policies & Laws	Government support and policies are needed to increase use and sustainability of sodium reduction initiatives.	• I think that this has to start not just in a household level, but I think, like the State, and the Government has to be way more committed to the health of our people, and obviously because they are there are already like contingencies and contracts in place is gonna be extremely hard for, like us to figure out like, whose responsibility is it. Because the government is going to say, Well, that’s the company’s responsibility and then the company is gonna say, well, if the government ain’t gonna put no sanctions on us we gonna do, we wanna do. (PIR 4, IDI). • So I think and I know there was initiatives…to like allow people to use WIC [Special Supplemental Nutrition Program for Women, Infants, and Children] at like farmers markets and like fruits and vegetable gardens and even get like a rebate or get like money back. But I just think more initiatives like that where it’s not just like low cost or ideally free, right for these healthier alternatives. (HP, FG 2)
Financing	Adequate funding for food and nutrition initiatives is lacking.	• But it’s hard too, right? For like the food banks and for shelters and things like that because they’re not operating on a really big budget. So that’s, that’s what’s tricky. And that’s where I think you need like the policy piece to support it. (HP, FG 2)
External Pressure	There is a disconnect between business profit-driven motives and consumer health.	• But ultimately, I think we feel to realize that those that are in business cannot, could not care less about what’s on these packages or whatnot. Their end goal is to make money. (PIR, FG)
Inner Setting		
Communications	Faith-based organizations and the health ministry (Pastors4PCOR) provide high quality information sharing practices.	• I’m part of the Health Ministry. Sky high, their blood pressure sky high, be like its okay, what did you eat today? And when they say, oh, I had a bag of chips, and then I had a pickle, and I had a can of pop. I’m like, okay, look, look at all this salt…you had a whole lot of sodium right there that you wasn’t necessarily aware of but it was sodium intake. (RMA3) • It’s almost the same thing I feel like I’ve heard about when you wanna work out like getting a workout, but be like just not being in something specifically new or not being alone is very, very helpful, like you have the teamwork aspect, and you have somebody to check you… (PIR, FG1)
Culture	Salt carries spiritual symbolism and is part of deep-rooted cultural traditions.	• When it comes to faith, faith-based communities…what Scripture says, I believe that ‘ye are the salt of the earth.’ And if that is the case, and if we are the salt of the earth, then there should be some kind of, I think, as far as faith-based communities are concerned, some kind of preservation or, yeah, some kind of preservation that should be accompanied with, you know, being a Christian and carrying the banner of Christ. (PIR 6, IDI) • I was thinking about it, even just the two references that we brought up talking about like being the salt of the earth, but also how Lots wife was turned to a pillar of salt? It’s like the connotations are different…I believe salt has a place, you know, in the faith-based community. It’s just going to depend on the context, on the environment, on the usage. And then I like the word that [participant] used that balance sort of thing. (PIR, FG1) • So I know in faith-based communities, they’re like a lot of meals. They’re like conferences and things of that nature. So food is always involved in community spaces. And it’s good food generally. So like, there’s a lot of salt that’s used for that. (PIR, FG1)
Compatibility	The COMBI-SR program fits within (healthcare) workflows, systems, and processes.	• I think the conversations should start before the provider enters the room. And that’s really looking deeper into like social determinants of health that’s usually acts by. You know the care team, whether it’s the nurse, the medical assistant, the dietitian, case manager…So when the provider goes into the room, being a provider, would know, you know, what’s the greatest, what are the greatest barriers or where to kind of focus? You know, provide kind of focus, support. So that’s just for me, I think that really, providing that appropriate workflow because a lot of times you can ask the questions. (HP, FG3)
Relative priority	Sodium reduction is often viewed as less important than other diet, health, and life concerns.	• Usually people have many other disorders that we’re trying to treat at the exact same time. And also like priority, to be completely honest. Like again, if there’s like depression or substance use disorder, like I have to go with what’s, you know, going to, I guess mostly like kill the patient first, it’s not the best way to put it…Even though I get it, diet is really, really important. It’s just, we’ve got a lot of things to cover in a really short amount of time. (HP, FG1) • And to be honest [I think] there is more [to] sugar….Most people don’t look past that very first line [calories], and it’s even strategic, and how they even set it up like labels, cause you’ll have the calories in big old [font]. But then, when you get down to sodium and all these different preservatives it’s all in very fine bent lines, so you can’t. (PIR, FG1)
Mission alignment	The evolving health focus among faith-based organizations and programs could integrate sodium reduction programs.	• Because like P4P, and like TRCDO, offers a lot of like health literacy classes and opportunities for engagement. They just had [something] like Man Church, where, like people came out, talked about like prostate cancer, and all of us like health stuff, mental health, and all this other stuff. And then they did the same thing for the women. And we try to like meet the needs. (RMA 4, IDI) • Our pastor is big on health as well. So he’ll be like. No, we still need to be like, he don’t eat a lot of stuff, but he’d be like, no, that’s not healthy. (RMA 4, IDI)
Available resources	Resources within the community to implement and deliver sodium reduction interventions are limited.	• I would say Whole Foods. But you know they just recently moved the Whole Foods…They shut it down and a lot of people I mean, the community is upset. When Whole Foods went to that part of [the neighborhood] it was a big thing, because Whole Foods is coming to the South Side, and then Whole Foods is going to Inglewood like what? That’s how we knew that the neighborhood…was on the brink of change. But now that Whole Foods is gone? They were trying. (PIR6)
Access to knowledge and information	Many community residents have limited knowledge on the amount of salt/sodium in food and the consequences of high sodium consumption.	• Salt is in everything if you’re eating bread, salt in it - You can be drinking your coffee, and your creamer has salt in it - like you actually don’t know what has salt in it. Or the sodium content of it. And I don’t think that we, as African American consumers, actually look at that part of it…Even our seasonings it could be. It doesn’t have to be seasoning salt, but 9 times out of 10, it has some type of sodium content. (PIR1) • But it is education. In that sense they become aware of that. These are not just numbers, and this is something that can affect your life overall. So educating is, education is a key, key aspect of oh, and following up on that is also another key aspect before they have a stroke or counter type, or anything like that, you know. (HP, IDI2)
Individuals		
Need (Innovation Recipients)	Community members are acutely familiar with many health conditions stemming from behaviors such as high sodium consumption.	• I don’t know the extent of the ailments and the diseases that plague my community, but I know that it’s a lot of ‘em and I know that a lot of them have to do with not having healthy options (PIR 6, IDI) • You know, in my family we have strokes and high blood pressure, you know. I don’t want to experience that. So that has been controlled, as you know, knowing myself and my body and my health you know you know what you can and cannot eat that may trigger certain things in your body. If that’s gonna be harmful. (RMA 5, IDI)
Capability (Innovation Recipients)	Many community members currently lack the knowledge and skills to reduce their salt consumption and read nutrition labels.	• “Salt intake cannot be controlled.” I disagree with that. I disagree with that, especially if you cooking. If you, if you preparing your own meals right, we control what we put in preparing those meals so yeah, it can be controlled if we prepare our own meals. Right? Now, when we eat out, you know, not so much. You know we don’t. We can’t control that. You know, we out but we definitely can control what we are prepared (PIR 3, IDI) • But I know now I need to still be educated on how to read these labels. What do they mean? But right now, if I see something like this, I’ll just stay away from, because in my mind I don’t need to eat that. I don’t need to drink that because of the sodium. (PIR 3)
Capability (Innovation Deliverers)	RMAs and health professionals believe they can educate and influence community members’ dietary behaviors, with the right information and training.	• Personally, I feel like I am very convincing, and I think that I’m very like natural. I show up. I show up as myself everywhere…I think I’ll do very well at explaining why it’s important or how to use it especially if it’s like user-friendly. But I think if I learn how to work here and like am confident in using it and can I kind of test that myself, I definitely would be like, alright. (RMA 4) • Like I don’t think I’m proficient, I kind of have a few good tips that I try to tell patients. (HP, FGD 1)
Opportunity (Innovation Recipients)	Community members have the availability and power to participate in sodium reduction.	• I think it’s access and it’s finances. You know, a lot of our patients, even if they had the finances, they still would be going to fast food a lot and I don’t think they know, but the two of them together makes it much easier to access, you know, highly processed foods with a lot of sodium. (HP, FGD 1) • It may work, but couple of challenges that we have is, you know, most of the people are not very well familiar with the smartphone. Even now, you know, they have the basic phone, but not the smartphone or the apps. Many of them are not, especially, of course, the older age group that still has challenges, you know…for people who have a bad connection, you know, even when I try to do a telehealth visit with patients through the video to the Zoom, and many people are still not first of all, comfortable with it. Secondly, they’re not, they don’t have the best Wi-fi connection available, and thirdly, they’re not comfortable using it. (HP 2)
Motivation (Innovation Recipients)	Community members may find personal sodium reduction to be inconvenient, effortful, and less preferred.	• Throughout our daily lives we are eating and consuming and not really paying attention about the you know how much of what ingredient - any ingredient - is in anything that we eat. So I do believe that some people, most people, are not aware of how much salt they could potentially be consuming. (PIR 6) • I’m optimistic, and I’m grateful to be learning this information now, so that in my mind I can make the necessary changes, and I can discipline myself, although life can be happening like. I want to live long. You know what I mean. Life may be lifelong…so I have to take the discipline, and I have to take the responsibility to get the information, receive it and go from there. (PIR, FGD)
Motivation (Innovation Deliverers)	Individual differences in and lack of control over the effects of sodium on blood pressure decrease implementers’ likelihood to deliver sodium reduction interventions.	• But we don’t have a lot of other information…not everybody responds strongly to salt, like the link between high salt and high blood pressure, it’s very true for some people and not that much true for a lot of other people. And if I feel like people don’t have that much control over it, I don’t emphasize it too much, just the reality of my own practice right now…it’s not one of the first things I go to. (HP, FGD 1) • I think that if they tasted [salt substitutes], like if there was a taste test and they could see that it tastes pretty much the same…I always push people to use it, but I think that they’re scared of it kind of because they’re not used to it. (HP, FGD 2)
Implementation Process		
Planning	Suggestions for the initial steps needed to implement sodium reduction interventions in the community.	• I think the first activity is education we have to be educated. We have to be educated on the harms. I mean, we know, but we don’t know. Once we know, we gotta start doing something about it…Now let’s have a health ministry. Let’s have a walking ministry. Let’s go out. If you go out to evangelize, you know. Let’s get these steps in. (RMA1, IDI) • But I would definitely say to start off with plain research. Really doing a deep dive into what we’re eating while we’re eating it? Where is it coming from? Who is making it? What is the pro? What is their process and making it and researching like where their actual labs are and where their farms are…see what comes with the food like like, what? What are they spraying on [it]? The main thing we need to do is like, have communities of research. (PIR, FG)
Tailoring strategies	Suggestions to choose and operationalize strategies to address barriers, leverage facilitators, and fit context.	• Yeah, if you could get people to think goals. Sometimes maybe weight goes. Sometimes blood pressure goes or a goal to say, Okay, I’m gonna work out 3 days a week for 15 min…they’ll feel proud of themselves and maybe want to continue (RMA3, IDI) • And we just have to stress that we need to live and not just live, but live and flourish and be happy and be healthy. You know I don’t just wanna live dormant. I wanna live positive. You know, I wanna be able to see my grandkids kids. (RMA1, IDI)
Engaging (Recipients)	Suggestions to attract and encourage community members participate in sodium reduction interventions.	• Activities. More health fairs, you know. I know we went virtual, everything online, I think, having more health fairs, educating the community especially in the Black community and then give and you know, educating and also providing resources, maybe giving people blood pressure cuffs, you know. Yeah. (PIR, FG) • I think we definitely need like huge marketing. So it’s just not like the people in my community. I think people to people in the community where my church is located should be invited to this, something like billboard like, let’s, do you know? Like, let’s do something, or like (PIR, FG)
Engaging (Deliverers)	Suggestions to attract and encourage healthcare providers and faith-based leaders to deliver sodium reduction interventions.	• I think you would need a counselor…It’s a mental thing of why you eat what you eat, why you eat much of it. Oh, you know what I’m saying when you come to salt, how much intake do you think you eat daily? So you need those types of questions to me [via] to counselling. I would say, someone like a behavior specialist. (RMA5, IDI) • First and foremost, the pastor, the head, of the household, the head of the church, you know it has to come from him [then] through the other leaders, through the rest. Once he’s vocalized it and spearheaded. Because let’s be real a lot of times, you know it doesn’t…it has to be from the top down, going down, and sometimes the bottom up is good, too, because that’s that’s young minds coming up talking about it. But in the beginning it has to be coming from the forefront. You talk about a church community, or a coach talking to his players, or whatever. But it has to be the leader. (RMA1, IDI
Doing	Suggestions for small steps to test and optimize delivery of sodium reduction interventions.	• Once you got the education you have…to do something about it. We have to study it, practice it, and then we teach it to other individuals, then it gets in your DNA, and it becomes a habit, and once it becomes a habit, it becomes a lifestyle. And then that’s when we can change and be healthier. (RMA1, IDI) • That there are certain foods that we eat when you, for for instance, me and my wife, we went on like a Vegan diet right? And then we came off the weekend. I think we did it for like a month we came off of it…it was almost like we our palettes were cleansed and we were seasoning stuff like how we always done it…And so I do think that once a palette is clean once you start to eat different foods it’s almost like your body trains itself or recalibrates itself and shows you what’s not normal (PIR, FG)
Adapting	Suggestions to make modifications to the innovation and context to fit sodium reduction interventions into the community.	•…a lot of times a church or a community setting, that’s what we feel most comfortable. We really don’t like going to the doctor. It’s not good sneezing, but somewhere where you go every Sunday or one day a week for Bible study, or a choir on a Tuesday night - you have somebody there where you already comfortable with this setting. (RMA1) • But that 5.5 g a day, I think even though, like I’m not gonna measure I think. But hearing that will make me - like will make me adjust. And also, like as I tell people like, “Hey, it’s as simple as when we have the saltshakers don’t shake so many times.” You maybe just shake once or twice, and that’s the practical-ness of that, that’s translating it in a more practical sense than say 5 g like a teaspoon cause no one, no one’s gonna really care too much about that… (PIR, FG)

Abbreviations: CFIR. consolidated framework for implementation research. COMBI-SR. Communication for behavioral impact for sodium reduction. FG. Focus group. HP. Healthcare professional. IDI. Individual interview. PIR. Potential intervention recipient. RMA. research ministry ambassador

#### Innovation

Innovation is the evidence-based “thing” being implemented—here, the innovation is the SaltSwitch app (individual level) and COMBI-SR messaging (community level).

Regarding *SaltSwitch*, participants noted the need for a user-friendly app with straightforward instructions and language to make the app accessible: *“It’s gotta be something they can access easily. And it be just plain*,* simple language” (RMA3)*. Participants preferred tools that enhance health consciousness and simplify nutrition decision-making, aligning with their current use of fitness and health-tracking apps. Moreover, participants expressed interest in adopting SaltSwitch if it is free or more affordable, provides real-time lower-sodium alternatives, and helps interpret food labels. While SaltSwitch was well-received for offering easy access to nutritional information and providing healthier alternatives, participants recommended improving its usability, particularly in selecting the best healthier alternatives. They emphasized that to engage a wide range of users, SaltSwitch must offer tasty, lower-sodium alternatives at affordable prices.

Concerns were raised about underlying difficulties with reading food labels and confusion about sodium content: *“The way nutritional information is written is confusing. You might think the listed amount is for the whole item*,* but it’s often for a single portion. A box might have 7 or 8 portions*,* so you have to multiply to figure out the total salt content” (PIR5*,* IDI).* While some found the dietary messages of *COMBI-SR* to be clear and effective, others felt that overly complex information could create resistance. Participants also responded positively to COMBI-SR’s public health message examples appreciating vibrant design and clear depictions (e.g., ‘5 g’ as one teaspoon).

Participants suggested including more information about the link between sodium intake and heart disease to better understand its relevance to their health. In fact, many potential intervention recipients and RMAs raised concerns about the sufficiency of available research on the role of salt intake on conditions like high BP, and suggested the need for more research: *“As it relates to biology as it relates to food*,* we still need a hundred more*,* a hundred to 400 more years to really speak accurately about like what salt is really doing in someone’s body” (PIR4*,* IDI)”.* Participants desired clearer explanations about salt intake recommendations, greater transparency in research methodologies, and more inclusivity to ensure sodium intake guidelines are accurate and relevant for diverse groups.

#### Outer setting

Outer setting is the context in which the Inner Setting exists—here, Outer Setting includes community/neighborhood factors (outside the faith-based community) driving implementation of sodium reduction interventions in Chicago’s South Side.

Participants indicated that use of salt, and thus sodium intake in the community, was deeply ingrained in the community. They related use of salt to gatherings, family, and building a sense of community through food: *“Salt*,* to me*,* symbolizes coming together and family. When it’s sprinkled on food*,* it enhances the taste and brings comfort*,* often drawing more people to gather around during events*,* gatherings*,* or meals” (RMA1*,* IDI)*. This same participant reflected on the deep-rootedness of salt intake, noting that it has become a generational habit, where food is automatically seasoned with salt without even tasting it.

Structurally, participants described the impact of living in food deserts on the community’s diet: *“There is no food places*,* not even McDonalds. So*,* this isn’t just a food desert for anyone who don’t have a transportation. It’s almost impossible to buy food from outside unless they cook food of their own*,* you know” (HP*,* FG2).* A potential intervention recipient described the pervasiveness of sodium in daily products: *“I feel like even my toothpaste has salt in it. It makes me wonder what’s really going on. Are we being set up for challenges in our communities and lives? It feels like we might have been sold a lot…” (PIR*,* FG).* Other participants noted closures of stores with healthier options (e.g., Whole Foods) but acknowledged opportunities in the rise of health-conscious dining options and the expanding availability of organic, farm-raised, and pesticide-free products.

Participants highlighted community organizations as key partners for promoting healthy eating. They suggested integrating healthy food options into shelters, distributing fresh produce through food pantries, and providing nutrition education through schools, FBOs, clinics, and community kitchens to support healthier dietary practices. While support for healthier eating exists, participants noted that many businesses still prioritize profits over nutrition.

Participants noted the importance of policies and community incentives to promote healthy eating and address food insecurity. They suggested expanding the Special Supplemental Nutrition Program for Women, Infants, and Children (WIC) to include sources like farmer’s markets and promoting healthier choices through discounts and rebates.

#### Inner setting

Inner Setting is the setting in which the innovation is implemented (e.g., hospital, school, city)—here, the Inner Setting includes the FBOs and community organizations who would be responsible for implementing COMBI-SR in Chicago’s South Side.

Within the faith-based community, potential intervention recipients and RMAs commented on the multifaceted role of salt in faith-based communities, highlighting its dual symbolism in religious and cultural contexts. They cited both positive (e.g., “salt of the earth”) and negative (e.g., Lot’s wife turned to salt) biblical references. Participants also described deep-rooted cultural traditions surrounding the use of salt in communal meals and noted resistance to changing traditional, generational recipes.

All participants emphasized the strength of the community networks within FBOs and their external partnerships in supporting health management. They highlighted efforts to promote health issues (e.g., high BP, headaches) via regular medical follow-ups, community health metrics, and support networks, including the Health Ministry: *“I’m part of the Health Ministry. Sky high*,* their blood pressure is sky high*,* and they act like its okay. So*,* I ask*,* what did you eat today? And when they say*,* oh*,* I had a bag of chips*,* and then I had a pickle*,* and I had a can of pop. I’m like*,* okay*,* look*,* look at all this salt…you had a whole lot of sodium right there that you weren’t necessarily aware of but it was sodium intake” (RMA3).* Other key communication channels for promoting healthy diet practices included online support groups and family discussions.

RMAs highlighted the shift among Pastors4PCOR and individual FBOs from a focus on sugar to embracing broader health initiatives, noting that sodium intake would be aligned with the overarching commitment, purpose, and goal of their community: *“Our pastor is big on health as well. So*,* he’ll be like…no*,* that’s not healthy” (RMA4*,* IDI).* As the RMAs described, the community “offers a lot of like health literacy classes and opportunities for engagement” (RMA4, IDI) with events like “Man Church” that addressed prostate cancer and mental health. Nonetheless, they also noted struggles with maintaining long-term engagement in health initiatives, citing a sugar reduction program that failed to sustain participant interest despite initial enthusiasm. Health professionals and RMAs also noted that, compared to other health concerns, dietary concerns and programs such as COMBI-SR were less critical: *“Usually people have many other disorders that we’re trying to treat at the exact same time…Even though I get it*,* diet is really*,* really important. It’s just*,* we’ve got a lot of things to cover in a really short amount of time” (HP*,* FG1).*

Participants cited insufficient education on the consequences of sodium consumption, particularly the extent of its presence in food and associated health implications, with one health professional stating, *“So educating is*,* education is a key*,* key aspect of oh*,* and following up on that is also another key aspect before they have a stroke or other type*,* or anything like that*,* you know” (HP*,* IDI2).* Lack of financial backing was cited as barrier to dietary program sustainability, nutrition class access, and effective health education and nutrition management broadly. Healthcare professionals noted the absence of targeted salt reduction programs and the difficulty of delivering comprehensive dietary education during brief clinical visits.

#### Individuals

The Individuals domain refers to the roles and characteristics (Need, Capability, Opportunity, Motivation) of individuals involved in implementation—here, we focused on Innovation Recipients (e.g., patients, community members) and Innovation Deliverers (e.g., RMAs, healthcare professionals).

Potential intervention recipients understood that negative health consequences, including hypertension, “plague their community” and that they have control over their salt intake, to a degree: *“if you preparing your own meals right*,* we control what we put in preparing those meals so yeah*,* it can be controlled if we prepare our own meals. Right? Now*,* when we eat out*,* you know*,* not so much…But we definitely can control what we prepared” (PIR 3*,* IDI).* Participants mentioned that some factors that limited individuals’ opportunity and motivation to engage in sodium reduction behaviors included lack of access to healthier food options, lack of finances to purchase healthier food options, and lack of information or knowledge.

Healthcare professionals and RMAs expressed varied levels of confidence in their ability to educate and positively influence their community members’ dietary behaviors. Whereas an RMA shared, *“Personally*,* I feel like I am very convincing*,* and I think that I’m very like natural. I show up. I show up as myself everywhere…I think I’ll do very well at explaining why it’s important or how to use it especially if it’s like user-friendly…” (RMA4)*, a health professional stated, *“Like I don’t think I’m proficient*,* I kind of have a few good tips that I try to tell patients” (HP*,* FGD1)*. Healthcare professionals further noted that their motivation to address dietary sodium among their patients was affected by the variability in individual biological responses to salt and the perceived lack of control over its effects on BP.

#### Implementation process

Implementation Process includes the activities and strategies used to implement SaltSwitch and COMBI-SR in Chicago’s South Side.

To prepare the community for implementation, all participants recommended various forms of education as the first step in promoting healthier lifestyles within their communities, consistent with the above barriers concerning gaps in knowledge about sodium among community members: *“I think the first activity is education*,* we have to be educated. We have to be educated on the harms. I mean*,* we know*,* but we don’t know. Once we know*,* we gotta start doing something about it…Now let’s have a health ministry. Let’s have a walking ministry. Let’s go out. If you go out to evangelize*,* you know. Let’s get these steps in” (RMA1*,* IDI).*

Participants emphasized that strategies should focus on positive, future-oriented messaging for community members. For example, one participant suggested a focus on promoting individual’s self-efficacy: *“if you could get people to think goals. Sometimes maybe weight goals. Sometimes blood pressure goals or a goal to say*,* Okay*,* I’m gonna work out 3 days a week for 15 min…they’ll feel proud of themselves and maybe want to continue (RMA3*,* IDI)*. Participants also valued visual learning, as demonstrations showing the hidden amounts of salt in foods will make the impact of education on dietary choices more effective. They expressed dissatisfaction with unclear information about sodium and advocated for straightforward, actionable messaging—such as “don’t shake [the saltshaker] so many times” (PIR, FG)—rather than abstract measurements (e.g., grams/day). Participants also advocated for implementing community-based programs in familiar environments (e.g., churches), which may be more welcoming environment: *“…a lot of times a church or a community setting*,* that’s what we feel most comfortable. We really don’t like going to the doctor.…somewhere where you go every Sunday or one day a week for Bible study*,* or a choir on a Tuesday night - you have somebody there where you already comfortable with this setting” (RMA1)*. Moreover, participants indicated that any behavior change approaches work to ensure that sodium reduction becomes internalized, practiced, and shared. Specific suggestions included: increasing physical activity and promoting an overall healthier lifestyle; receiving personalized health advice from physicians through direct discussions about health; and modifying diets to recalibrate taste preferences.

To effectively engage community members in sodium reduction programs, participants emphasized that effective leadership in dietary education and change should start with professionals who can address the psychological aspects of eating habits, such as counselors or behavior specialists, as well as pastors and other formal or informal leaders. As one participant stated, *“First and foremost*,* the pastor*,* the head*,* of the household*,* the head of the church*,* you know it has to come from him [then] through the other leaders…in the beginning it has to be coming from the forefront. You talk about a church community*,* or a coach talking to his players*,* or whatever. But it has to be the leader” (RMA1*,* IDI)*. They also recommended a variety of activities (e.g., health fairs, cooking classes) and modalities (e.g., in-person, virtual) to engage a larger swath of the community,

### Strategies for implementing COMBI-SR

Implementation strategies refer to the methods or techniques to enhance the implementation and sustainability of an innovation. To successfully implement COMBI-SR in Chicago’s South Side, participant responses (healthcare professionals only) were coded into 20 strategies from the ERIC compilation (Table 4 and Supplemental Material 2).

**Table 4:** Summary of identified strategies, per the expert recommendations for implementing change (ERIC) compilation, for implementing the COMBI-SR program

ERIC Cluster and Strategy	Summary of themes	Illustrative quotes
Use evaluative and iterative strategies		
Conduct local need assessment	Collect and analyze data related to the need for sodium reduction interventions	• I think if you have buy-in from executive leadership, and the way you do that is by showing them the numbers. Like, I know our hypertension numbers are horrible; they’re abysmal. Someone may not be—I don’t want to be morbid—but it may not be tomorrow. But this is a big problem, and it’s going to really negatively impact our patients. So, I think getting buy-in by showing them the impact is crucial because most people are there because they care about their patients. And a lot of our patients face many different challenges in their lives. So, something like that would be critical. • I think again, like, if you have the resources to understand, like to spend the time and understand, like what people specific barriers are, then you could target them.
Adapt and tailor to context		
Tailor strategies	Tailor the implementation strategies to address barriers and leverage facilitators that were identified through earlier data collection	•…tailoring the plan through a team approach like with the nurse. And you know, maybe like a little breakout like kind of nutritionist in the office if there is one. So yeah, tailoring it, individualizing it.
Promote adaptability	Identify the ways sodium reduction interventions can be tailored to meet the community’s needs	• I think that’s hard because sometimes it’s just a lot of it is like geographical…So something that’s like accessible to one group of people. Like I could do the whole city, so it’s not reasonable for a different part. • But then again, they need to figure out if they’re waiting for people who are going to have dramatic changes in salt intake before knowing whether or not they need to commit to that. So this is why it’s a little bit harder. And yet for those one in four, it makes a huge difference. So I say try to cut it down, here’s ways to cut it down, see if it makes a difference.
Develop partner interrelationships		
Identify and prepare champions	Identify and prepare individuals who dedicate themselves to supporting, marketing, and driving implementation of sodium reduction interventions	• For getting community leaders involved, there’s going to have to be some type of probably incentive for them to do it as well. And I think repetition is key too, just like talking about it all the time, you know, bringing it up. Even if it’s not the most important thing that they’re there for that day…I don’t know how important hypertension is when there’s like shootings going on and all that stuff, you know? So I guess, like I said, again, repetition and community education would be probably the best things. • You have to interview them and make sure that they’re the right fit for your program. Because if you’re just trying to hire somebody just to get the survey done, then I don’t know, it’s not going to really work. Because, you know, patients need to know that you care for them so they can care for themselves sometimes.
Build a coalition	Recruit and cultivate relationships with partners who will support implementation of sodium reduction interventions	• The South side has the highest net density, like more churches than any other community in the US per population. So going into the churches which are trusted spaces to work with pastors, to work with church kitchens, thinking about where are the places where people come together to cook together…The main distributors of food knowledge would be where communities come together to eat and figuring out where those spaces are. • Work with CPS [Chicago Public Schools] to make sure that there are salad bars in the schools like structurally, is each school able to serve low sodium food or there is one high school on the south side where they didn’t have a school kitchen, it was a charter school. So they literally served cold cut sandwiches every day and they couldn’t have a solid parks, there was no place to have it. So just making sure that some of the institutions that are government funded that touch everybody in the community are able to start sharing that knowledge early. • The community gardens too. So community gardens, school gardens, all that growing food. So if you want to even take it a step back, there is a backup plan to continue the initiative.
Capture and share local knowledge	Capture local knowledge from organizations that made similar interventions work in their setting	• But in terms of how to operationalize cooking classes, there are some health centers that have kitchens that can be tapped into as local spaces. So to identify spaces that have kitchens that you can then use for the cooking demonstrations, where you do a demonstration that everyone can sit down and eat together so that you go, you cook and you get a good meal from it. • Feeding America…they’ve done a huge like change of like, like what can be like donated and all these things because usually, you know, for lack of professionalism, we just give our crap, right? But now there’s been a huge change in that and I think it’s slowly trickling down.
Train and educate implementation partners		
Conduct ongoing training	Plan for and conduct training in sodium reduction in an ongoing way	• But like again, the science of this, I would really, we love to see the science of what’s the best approach. • I was like, apparently you need to retrain providers.
Conduct educational meetings	Hold meetings for different groups in the community to teach them about sodium reduction	• I mean, there is different programs. One is for any patient we give like fruits, vegetables and all those but the shop printing, and all these are mainly specifically for what they can eat. And you know, and what do you need to buy when they go to the store? And all those things. So it’s really about one on one education. And right, kind of shopping for the food for the phone.
Support clinicians		
Revise professional roles	Shift and revise roles among healthcare workers to accommodate a sodium reduction intervention	• It’s really it’s just getting to know all the patients and having people be made aware. I try to communicate with the MAs a little more and kind of get, you know, get them on board to kind of help out if we have time, you know, with those kinds of things. I’m really, really impressed with our teams, you know, and I think that they would be up for more of that.
Create new clinical teams	Change who serves on the clinical team, adding different disciplines and different skills to make it more likely that the sodium reduction intervention is delivered	• What about getting some student volunteers from social work programs to come help, you know, to volunteer? Like they get something, we get something, they have some accountability, right?…Or nurses from nursing school? • Maybe it is like a worker or somebody like a role that does like, looks for all the resources and then like provides them to, like, I’m just thinking about how to like disseminate that, um, by area or something like that.
Engage consumers		
Involve patients/consumers and family members	Engage or include community members and their families in the implementation effort	• In the pharmacy I do the same thing too. Like after when I’m counseling patients, I’m constantly giving them handouts and even if they don’t read it, I feel better that I gave it to them. And maybe next time when they come in, we can talk about it or something like that. Or sometimes I give them homework…So I give them assignments like, you know, next time you come in, you better tell me the top five things on there…Yeah, handouts and visual aids are super helpful. • And then I think, another thing that’s really important is not only educating the patient, but figuring out what their living situation is so like. Maybe that person wants to eat less sodium. But everyone else in their house does not so like understanding that. And you know, getting family or people who are living with them kind of on board and understanding, you know, like the whole unit as opposed to just the one person.
Intervene with patients/consumers to enhance uptake and adherence	Develop strategies with community members to encourage and problem-solve around adherence	• And I thought about this like just literally meeting them where they’re at like what’s your ability to go to the grocery store? Are there any grocery stores around you, or do you just have a convenience store kinda like nearby, you know, you went and grabbed a pizza, you know, at this place like literally. • Many things can be addressed by being in the community rather than the clinic reaching out to, you know. We have a good community outreach program where they, where we have events to teach them cooking. We are planning to build a nutrition center attached to this clinic where the community can come and eat. Of course they have to pay the money, but healthy food choices are expensive. And my dietician here she takes the people. She’s a diabetic educator. She takes the patients out for shopping in one of the local grocery stores. It’s called Market where we have an arrangement with them that they give healthy food for like foods, vegetables, all those healthy foods at current use cost to the patient. So we do such a outrageous. And then also we get donations of healthy green leafy vegetables and fruits and vegetables doing certain fruit store, I mean full store, which we donate to our patients, too. So those, I think, and things can really help for them to be more eat, more healthy food.
Prepare patients/consumers to be active participants	Prepare community members to be active in their health, health care, and health behaviors, particularly related to sodium reduction and cardiovascular health	• And eating habits are formed young. They are really difficult to change when you’re older, really difficult. So if you grow up on your healthy snacks, more likely you’re going to crave those later. But if you just get diagnosed with X, Y, Z, and then you have to change all your eating habits, that’s a big ask. Like that’s really, people can do it, but that’s a bigger ask. So I totally agree. Get it in the schools, we’re already having to provide food for schools, so do it. And I say make it like whatever, like the gardens, all that good stuff. There’s so many schools that do that now. • The other thing that I’m just thinking culturally is, because it’s a question of do you have access to your own kitchen? If you do have a kitchen, do you have the pots and pans you need to cook for yourself? If you do have the pots and pans, you need to cook for yourself, and the oil and the basic ingredients, do you know how to put them together to make food? Has this been taught? Has this been trained? Does it get handed down from parents to children? Has there been that knowledge dropped? And if so, how do you recreate the knowledge of how to cook while having the basic supplies necessary to cook rather than eating the processed foods and the fast foods
Use mass media	Use media to reach large numbers of people to spread the word about sodium reduction	• And some people don’t even realize anything like we know about salt, they don’t think about that at all. So I think having like, a whole campaign dedicated to that, um, advertising and all that.
Utilize financial strategies		
Fund and contract for the clinical innovation	Identify government programs and other payers of services to support implementation of sodium reduction interventions	• So I think and I know there was initiatives, I don’t know if they’re still in place to like allow people to use WIC [Special Supplemental Nutrition Program for Women, Infants, and Children] at like farmers markets and like fruits and vegetable gardens and even get like a rebate or get like money back. But I just think more initiatives like that where it’s not just like low cost or ideally free, right for these healthier alternatives, but that there also is like an incentive like, oh man, I’m not just going to get this for free, I’m going to get this extra thing. Yeah. So I think just working with, you know, different kind of. But it’s hard too, right? For like the food banks and for shelters and things like that because they’re not operating on a really big budget. So that’s, that’s what’s tricky. And that’s where I think you need like the policy piece to support it.
Access new funding	Access new or existing money to facilitate implementation of sodium reduction interventions	• But again, it needs to be funded, it needs to be seen as health care. It needs to be funded just as much as like big pharma, you know, because it’s just as, it’s more important but yeah. • Everything that happens needs to be funded through a grant or something because, you know, the clinics are barely trying to keep their doors open. And, you know, they have a lot of patients who are self-pay who really don’t get paid for their thing. So, you know, we would have to focus on or you guys would have to focus on finding grants for all of these extra stuff you guys would like to add.

Note. The ERIC cluster of “Provide iterative assistance” was not coded in the available data

Abbreviations: COMBI-SR. Communication for Behavioral Impact for Sodium Reduction. ERIC. Expert Recommendations for Implementing Change

Healthcare professionals mentioned that an important strategy for implementing COMBI-SR includes continuous refinement of implementation efforts through *evaluative and iterative strategies*, particularly by conducting local needs assessments to collect and analyze data on sodium impact and sharing these findings with key executive leadership in government to gain their support. Participants noted the importance of *adapting and tailoring strategies* to local contexts, recognizing that each community has unique barriers and facilitators that must be addressed to ensure effective sodium reduction. Key strategies include securing buy-in from executive leadership in government, providing personalized, team-based care with nurses and nutritionists to address patients’ unique circumstances, and maintaining continuous patient engagement through individualized education. *Developing strong partner interrelationships* emerged as a key approach, with a particular focus on engaging community leaders, leveraging trusted spaces like churches, and partnering with local organizations such as “Feeding America” to build a coalition to reduce sodium consumption in the community. Participants highlighted that observing successful dietary interventions (e.g., community kitchens) at other sites in the community could provide valuable insights for COMBI-SR implementation. They also emphasized the need for ongoing training and education for key constituents, particularly healthcare professionals, to ensure they are well-equipped to support sodium reduction efforts (*Train and educate implementation partners*). Practical approaches such as offering food samples, conducting dietary recalls, and providing tailored shopping guidance were recommended to empower individuals to make healthier choices.

To *support clinicians*, participants suggested revising professional roles and creating new clinical teams, potentially involving medical assistants, social work students, and nursing students, to enhance team support and improve communication with patients. Recommended strategies for *engaging consumers* included community-level education, practical resources, and simple steps (e.g., how to read nutrition labels, avoiding extra salt) would help patients feeling overwhelmed by the abundance of information online. They recommended patient education campaigns, motivational interviewing, and food demonstration classes to enhance patient engagement with public health messaging and SaltSwitch. Healthcare professionals mentioned the need for funding and policy support, such as allowing WIC benefits to be used at farmers markets, to make healthier food options more accessible and appealing (*utilize financial strategies*).

## Discussion

This study explored the contextual factors in Chicago’s South Side and strategies for implementing COMBI-SR. Participants provided nuanced perspectives about the role of sodium in their lives and how to approach sodium reduction in this community, particularly through the lens of urban, faith-based communities. Our study aligns with previous research on COMBI-SR [12, 14, 15] and highlights the importance of adapting the intervention to the local context, addressing specific barriers, and leveraging community strengths to reduce sodium intake. Notably, while both Land et al. [15] and Do et al. [14] recognized the need for formative work and went so far as to engage community members to plan for COMBI-SR implementation, they did not publish the details of the contextual factors and strategies for adaptation. This study provides a comprehensive overview of the key determinants and strategies for adapting COMBI-SR for an urban, predominantly AA neighborhood in the U.S. The next step of this work is to test the effectiveness and implementation of the adapted COMBI-SR on sodium intake and BP of adults with hypertension in the community.

Our study contributes to the field of implementation science by showing how established frameworks such as CFIR 2.0 [18] and taxonomies such as ERIC [19] can guide the adaptation of nutrition interventions like COMBI-SR for specific populations. In the context of sodium reduction, our findings align with broader literature on nutrition interventions that emphasize the need for culturally tailored messaging and ongoing community engagement, especially in underserved communities where factors such as food access, trust, and cultural practices play a significant role in implementation success [25]. Digital tools for sodium reduction, like SaltSwitch, show promise but require careful integration with trusted institutions and culturally resonant delivery channels [26]. Further, our application of the CFIR 2.0 framework and ERIC compilation highlights both the utility and limitations of these frameworks in community settings. While CFIR 2.0 and ERIC are robust research tools, our experience suggests that successful implementation also requires iterative adaptation and co-creation with community partners to address context-specific challenges not fully captured by existing frameworks [27].

This work emphasizes the multifaceted role of FBOs and the integration of cultural and religious considerations into health promotion strategies. Integrating interventions into familiar, trusted community and cultural contexts, such as FBOs, can improve engagement [28]. Moreover, community-based programs can address gaps in sodium awareness and nutritional education and empower community members to make healthier choices [29]. Encouraging gradual, manageable sodium changes, alongside offering continuous support, is likely to result in long-term behavioral changes. This approach aligns well with established strategies for effective health promotion, enhancing potential for sustained sodium reduction and overall health improvement in the community [30]. By addressing these crucial factors, COMBI-SR messaging, the SaltSwitch app, and their implementation can be effectively adapted to meet the unique needs of South Side residents.

This study has limitations worth noting. First, we did not ask participants to directly link the noted contextual factors with specific implementation strategies, which may have provided a more comprehensive understanding of the COMBI-SR adaptation process. Second, we removed questions about implementation strategies from the potential intervention recipients and RMA interview guides. Although this decision was made to honor participants’ preferences and uphold principles of community-engaged research, it limited our ability to explicitly gather strategy suggestions from this key group. However, our analysis of the CFIR 2.0 Process domain is an adequate representation of community-informed potential strategies until future research seeks direct input on implementation strategies from intervention recipients. Finally, as the study was conducted in a single urban community, findings may not be generalizable to other settings.

## Conclusions

Our study identified unique challenges and opportunities to implement the COMBI-SR program in Chicago’s South Side, particularly in faith-based communities, and selected implementation strategies that may address the noted barriers. Next steps include conducting a large-scale clinical trial to evaluate the effectiveness and implementation of the adapted COMBI-SR. Our findings highlight the importance of culturally tailored, community-based approaches in health promotion and underscore the need for supportive policies that address the broader social determinants of health.

## Electronic supplementary material

Below is the link to the electronic supplementary material.

Supplementary Material 2

Supplementary Material 1

## Data Availability

All data produced in the present work are contained in the manuscript.

## Abbreviations

AA: African American
AHA: American Heart Association
BP: Blood Pressure
COMBI-SR: Communication for Behavioral Impact for Sodium Reduction
COREQ: Consolidated Criteria for Reporting Qualitative Research
CFIR: Consolidated Framework for Implementation Research
FBO: Faith-Based Organization
ERIC: Expert Recommendation for Implementing Change
FQHC: Federally Qualified Health Center
Pastors4PCOR: Pastors for Patient-Centered Outcomes Research
PCORI: Patient-Centered Outcomes Research Institute
RMA: Research Ministry Ambassador
U.S.: United States
